# Galpha_s_-coupled receptor signaling actively down-regulates α_4_β_1_-integrin affinity: A possible mechanism for cell de-adhesion

**DOI:** 10.1186/1471-2172-9-26

**Published:** 2008-06-05

**Authors:** Alexandre Chigaev, Anna Waller, Or Amit, Larry A Sklar

**Affiliations:** 1 Department of Pathology, University of New Mexico Health Sciences Center, Albuquerque, NM 87131, USA; 2 Cancer Research and Treatment Center, University of New Mexico Health Sciences Center, Albuquerque, NM 87131, USA

## Abstract

**Background:**

Activation of integrins in response to inside-out signaling serves as a basis for leukocyte arrest on endothelium, and migration of immune cells. Integrin-dependent adhesion is controlled by the conformational state of the molecule (i.e. change in the affinity for the ligand and molecular unbending (extension)), which is regulated by seven-transmembrane Guanine nucleotide binding Protein-Coupled Receptors (GPCRs). α_4_β_1_-integrin (CD49d/CD29, Very Late Antigen-4, VLA-4) is expressed on leukocytes, hematopoietic stem cells, hematopoietic cancer cells, and others. Affinity and extension of VLA-4 are both rapidly up-regulated by inside-out signaling through several Gα_i_-coupled GPCRs. The goal of the current report was to study the effect of Gα_s_-coupled GPCRs upon integrin activation.

**Results:**

Using real-time fluorescent ligand binding to assess affinity and a FRET based assay to probe α_4_β_1_-integrin unbending, we show that two Gα_s_-coupled GPCRs (H2-histamine receptor and β2-adrenergic receptor) as well as several cAMP agonists can rapidly down modulate the affinity of VLA-4 activated through two Gα_i_-coupled receptors (CXCR4 and FPR) in U937 cells and primary human peripheral blood monocytes. This down-modulation can be blocked by receptor-specific antagonists. The Gα_s_-induced responses were not associated with changes in the expression level of the Gα_i_-coupled receptors. In contrast, the molecular unbending of VLA-4 was not significantly affected by Gα_s_-coupled GPCR signaling. In a VLA-4/VCAM-1-specific myeloid cell adhesion system, inhibition of the VLA-4 affinity change by Gα_s_-coupled GPCR had a statistically significant effect upon cell aggregation.

**Conclusion:**

We conclude that Gα_s_-coupled GPCRs can rapidly down modulate the affinity state of VLA-4 binding pocket through a cAMP dependent pathway. This plays an essential role in the regulation of cell adhesion. We discuss several possible implications of this described phenomenon.

## Background

Integrins, one of the largest families of cell adhesion molecules play an important role in the regulation of immune responses and leukocyte traffic, development, hemostasis, and cancer [1]. A unique feature of integrins is in their ability to rapidly and reversibly regulate cell adhesion, and thereby modulate cell recruitment and homing, without a significant change in the expression of the molecule. Understanding the molecular mechanisms that regulate rapid changes in cell adhesion avidity is essential, since integrins are known to play roles in many human diseases. They represent an attractive target for several existing and emerging drugs for treatment of inflammatory diseases, anti-angiogenic cancer therapy, anti-thrombotic therapy, and others [2-5].

α_4_β_1_-integrin (CD49d/CD29, Very Late Antigen-4, VLA-4) is expressed on peripheral blood leukocytes, hematopoietic progenitors/stem cells, hematopoietic cancer cells, and others [6-8]. On circulating lymphocytes, VLA-4 has the potential to exist in multiple affinity states that can mediate tethering, rolling, and arrest on a ligand (CD106, Vascular Cell Adhesion Molecule-1, VCAM-1) that is up-regulated on inflamed endothelia [9,10]. The ability of leukocytes to arrest, extravasate, and navigate is directly related to the activation of VLA-4 integrin through Gα_i_-coupled GPCRs. Classical chemoattractants and chemokines that include formyl peptide and SDF-1 (CXCL12) are examples of Gα_i_-coupled GPCR ligands [11]. Gα_i_-coupled GPCRs activate integrin by triggering an inside-out signaling pathway. The change in the affinity for the ligand as well as the unbending (extension) of the integrin molecule are believed to be a part of this activation process [12-14].

We have developed a VLA-4 specific fluorescent probe that allows us to monitor VLA-4 affinity changes in real-time on live cells. Our model cell line is undifferentiated U937 cells, which do not attach to plastic surfaces nor exhibit homotypic adhesion [15], and constitutively express VLA-4 integrins in a low affinity state [6]. In these undifferentiated U937 cells VLA-4 affinity was rapidly and transiently triggered by several different Gα_i_-coupled receptors, and the kinetics of the affinity change reflected the desensitization kinetics of a particular GPCR [6]. Moreover, in a VLA-4/VCAM-1 specific cell adhesion system, the time course of avidity changes in response to receptor activation coincided with the time course of the affinity changes [16]. The increased cell aggregation could be accounted for by VLA-4 affinity changes rather than integrin clustering or increasing number of VLA-4/VCAM-1 bonds [17]. Using the same VLA-4 specific fluorescent probe, we have also developed a FRET based assay for the detection of VLA-4 unbending (extension) upon inside-out activation [18], which was validated in a series of publications [19-21]. We found that rather than being intrinsically related to the affinity change of the integrin binding pocket, the conformational unbending of the VLA-4 molecule is regulated by a different signaling pathway, in a different temporal fashion [20].

Recently, it has been proposed that in addition to well-known pro-adhesive signaling events (modulated by Gα_i_-coupled GPCRs), anti-adhesive signaling events can participate in regulation of cell adhesion, diapedesis and chemotaxis [11]. We noted previously that anti-inflammatory signals could be derived from Gα_s_-coupled signaling pathway [22], and recently that U-73122, a suspected histamine analog [23], could inhibit the VLA-4 affinity increase [20]. The goal of the current work was to explore whether the Gα_s_-coupled GPCR related pathway can affect the affinity or conformational state of the integrin. We found that two Gα_s_-coupled receptors constitutively expressed on U937 cells can actively down regulate VLA-4 affinity, after activation through two Gα_i_-coupled GPCRs. This affinity down regulation was not modulated by the change in the intracellular Ca^2+ ^concentration. Furthermore, the unbending of VLA-4 integrin, as detected using the FRET based assay, was not significantly affected by Gα_s_/cAMP signaling pathway. Using a VLA-4/VCAM-1-specific myeloid cell adhesion model system, we found that blocking the integrin affinity change by the activation of the Gα_s_/cAMP signaling pathway had a statistically significant effect upon cell adhesion. However, we also detected an activation of cell adhesion by Gα_i_-coupled GPCR under these conditions (when the affinity change was blocked). In the absence of the affinity change a rapid increase in cell adhesion was attributed to the unbending (extension) of the integrin molecule.

## Results

### Signaling through Gα_s_-coupled receptors blocks Gα_i_-coupled receptor-induced VLA-4 affinity up-regulation

The LDV-FITC probe, which mimics binding of a natural ligand to VLA-4, was used to study the effect of Gα_s_-coupled receptor signaling on the VLA-4 affinity change. Because the concentration of the LDV-FITC probe in these experiments (4 nM) has been chosen to be lower than K_d _for the resting state of VLA-4 and higher than the K_d _for the activated state, the transition from the low to the high affinity state was accompanied by additional binding of the probe. As shown previously, activation of Gα_i_-coupled GPCRs leads to up-regulation of VLA-4 affinity, and the LDV-FITC binding kinetics reflects the activation kinetics for a particular GPCR. LDV-FITC binding is rapid and transient for wild type receptors (because of the receptor desensitization), and rapid and sustained for a non-desensitizing mutant of FPR [6].

**Figure 1:**
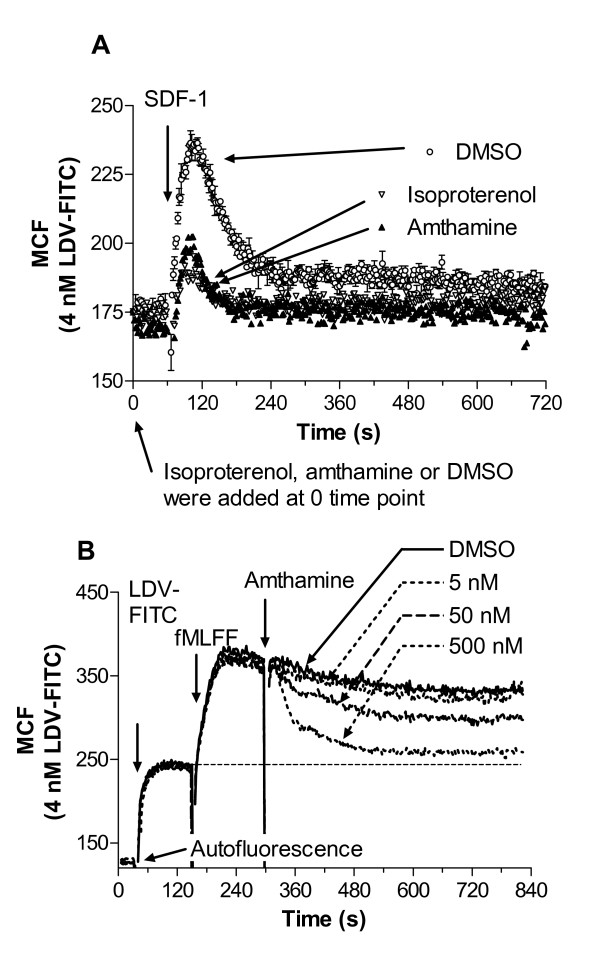
**Binding and dissociation of the LDV-FITC probe in response to Gα_s_-coupled receptor agonists**. Experiments were conducted as described under "Methods". A, LDV-FITC probe binding and dissociation on U937 cells stably transfected with CXCR4 receptor plotted as mean channel fluorescence (MCF) versus time. The experiment involved sequential additions of Gα_s_-coupled receptor ligands (isoproterenol (100 nM) or amthamine (500 nM)), then, fluorescent LDV-FITC probe (4 nM, below saturation, added 3 min prior to addition of Gα_i_-coupled receptor ligands). Control cells were treated with vehicle (DMSO). Next, cells were activated with SDF-1 (25 nM, arrows). Rapid and reversible binding of the probe reflects VLA-4 affinity change [6]. Curves are means out of two independent runs calculated on a point-by-point basis. B, LDV-FITC probe binding and dissociation on U937 cells stably transfected with the non-desensitizing mutant of FPR (ΔST) [33] plotted as mean channel fluorescence (MCF) versus time. The experiment involved sequential additions of fluorescent LDV-FITC probe (4 nM), fMLFF (100 nM), and different concentrations of amthamine, or DMSO (control) (arrows). The MCF value corresponding to cell autofluorescence is indicated by the horizontal arrow. One representative experiment out of three experiments is shown. Experiments shown in the different panels were performed using different instruments, and therefore MCF values are not identical.

To study the effect of Gα_s_-coupled receptors we took advantage of two Gα_s_-coupled receptors that constitutively expressed on U937 cells: histamine H2 receptor and β2 adrenergic receptors [24,25]. U937 cells stably transfected with CXCR4 receptor, preincubated with 4 nM LDV-FITC, were treated with receptor-specific agonists at saturating concentration (amthamine or isoproterenol) and DMSO (control). Next, cells were activated with CXCL12 (SDF-1). While control cells showed rapid and transient activation of VLA-4, cells preactivated through Gα_s_-coupled receptor showed a significantly lower response (Fig. 1A). Although, a slight decrease in the LDV-FITC binding to the resting cells after Gα_s_-coupled receptor stimulation was observed, amthamine or isoproterenol alone did not affect the resting VLA-4 affinity as measured by dissociation rate analysis (data not shown). The majority of integrin molecules on resting U937 cells exhibit low affinity (see Fig. 5 in [6]), however a small population of high affinity sites that cannot be detected by a dissociation rate analysis could exists. Gα_s_-coupled receptor signalling can eliminate this small fraction of active integrins.

The use of a non-desensitizing mutant of FPR allowed us to study the effect of Gα_s_-coupled activation in real-time. In the absence of Gα_i_-coupled GPCR desensitization, VLA-4 maintained its high affinity state for a longer period of time [6,19]. Addition of specific Gα_s_-coupled receptor ligands in this case led to a dose dependent down regulation of integrin affinity that was reflected in the rapid dissociation of the LDV-FITC probe (Fig. 1B). Thus, according to the analysis of LDV-FITC ligand binding, activation of Gα_s_-coupled GPCRs prevents VLA-4 affinity up-regulation, if done prior to Gα_i_-coupled receptor signaling, or it rapidly down regulates VLA-4 affinity, if performed during continuous Gα_i_-coupled receptor signaling.

### The effect of Gα_s_-coupled receptor agonists is reversed by specific receptor antagonists

**Figure 2:**
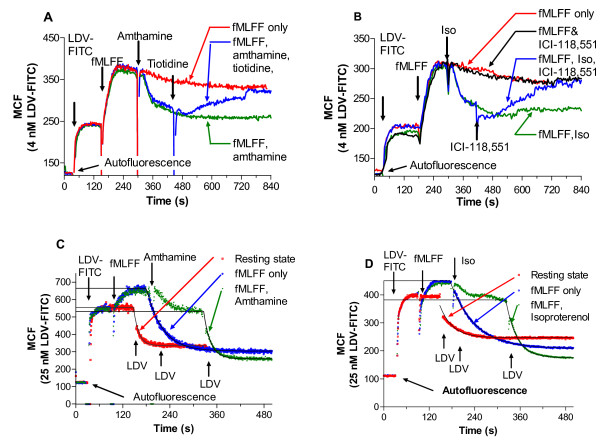
**Binding and dissociation of the LDV-FITC probe in response to Gα_s_-coupled receptor agonists and antagonists**. A, B, The effect of Gα_s_-coupled receptor antagonists. U937 cells stably transfected with the non-desensitizing mutant of FPR were sequentially treated with the LDV-FITC probe (4 nM, below saturation), fMLFF (100 nM), and A, amthamine (500 nM), tiotodine (10 μM), or B, isoproterenol (1 nM), ICI-118,551 (10 μM). B, Additional control sample was treated with the same concentration of LDV-FITC, fMLFF, and ICI-118,551 without addition of isoproterenol to show the effect of Gα_s_-coupled receptor antagonist without addition of the agonist. The MCF value corresponding to cell autofluorescence is indicated by the horizontal arrow. Data are plotted as MCF versus time. One representative experiment out of three experiments is shown. C,D, Kinetic analysis of binding and dissociation of LDV-FITC probe on U937 cells stably transfected with the non-desensitizing mutant of FPR. Cells were sequentially treated with the LDV-FITC probe (25 nM, near saturation), Gα_i_-coupled receptor ligand (fMLFF, 100 nM), Gα_s_-coupled receptor ligands, C, amthamine (500 nM), or D, isoproterenol (100 nM). At time points indicated by arrows, cells were treated with excess unlabeled LDV containing small molecule (2 μM), and the dissociation of the fluorescent molecule was followed. Dissociation rate constants (k_off_) were obtained by fitting dissociation curves to a single exponential decay equation (as described in the text). Experiments shown in the different panels were performed using different instruments, and therefore MCF values are not identical.

Next, to verify whether the observed effects are receptor-specific, we used two specific Gα_s_-coupled receptor antagonists: tiotidine (histamine H2 receptor antagonist), and ICI-118,551 (β2 adrenergic receptor antagonist) [26,27]. Addition of receptor antagonists after U937 cells were treated with Gα_s_-coupled receptor agonist completely reversed a suppressive effect of Gα_s_-coupled receptor signaling (blue lines, Fig. 2A,B). In this case, at the end of the experiment the binding of the LDV-FITC probe returned back to the level of the untreated control (red lines, labeled fMLFF only, Fig. 2A,B). It is worth noting that Gα_s_-coupled receptor antagonists by themselves did not have any effect on fMLFF activated samples (see Fig. 2B, black line labeled fMLFF, ICI-118,551), nor did they activate VLA-4 when added alone (data not shown). Thus, Gα_s_-coupled receptors provide a signal that results in VLA-4 affinity down regulation, and this effect is receptor specific since specific receptor antagonists reversed the effects of Gα_s_-coupled receptor agonists.

### Dissociation rate analysis confirmed that changes in the LDV-FITC probe binding could be attributed to the change in the affinity of the binding pocket

For different affinity states of VLA-4, the equilibrium dissociation constant (K_d_) of the LDV-FITC probe varied inversely with the dissociation rate constant (k_off_), suggesting that the association rate constant was essentially independent of receptor conformation [6,10]. Therefore, we routinely use dissociation rate analysis to quantitatively probe the affinity state of the VLA-4 binding pocket. Cells were preincubated with a higher concentration of LDV-FITC (25 nM) to saturate the majority of low affinity sites. Next, a large excess of the unlabeled LDV competitor is added to initiate a dissociation of labeled probe. After activation through Gα_i_-coupled GPCR, little additional binding of the probe was seen (since the K_d _for the low affinity state is ~12 nM (Table I in [6]), at 25 nM ~70% of sites are occupied before activation) (Fig. 2C,D). At rest, the majority of VLA-4 molecules present on U937 cells exhibit the low affinity state (k_off_~0.1–0.05 s^-1^). After Gα_i_-coupled receptor activation by fMLFF the dissociation rate was significantly slower (k_off_~0.01–0.025 s^-1^, slower k_off _indicates higher affinity). After additional stimulation through two different Gα_s_-coupled receptors, dissociation rates were comparable to the rate at the resting state. These k_off _values for the resting and activated VLA-4 receptor were quantitatively similar to previously published data [6]. Thus, the analysis of dissociation rates at rest, after Gα_i_-coupled receptor activation, and after Gα_s_-coupled receptor stimulation indicated that the Gα_s_-coupled receptor related signaling pathway is decreasing VLA-4 affinity by increasing the dissociation rate of the ligand.

### Gα_s_-coupled receptor activation has no significant effect upon expression of Gα_i_-coupled receptors and VLA-4 integrin

**Figure 3:**
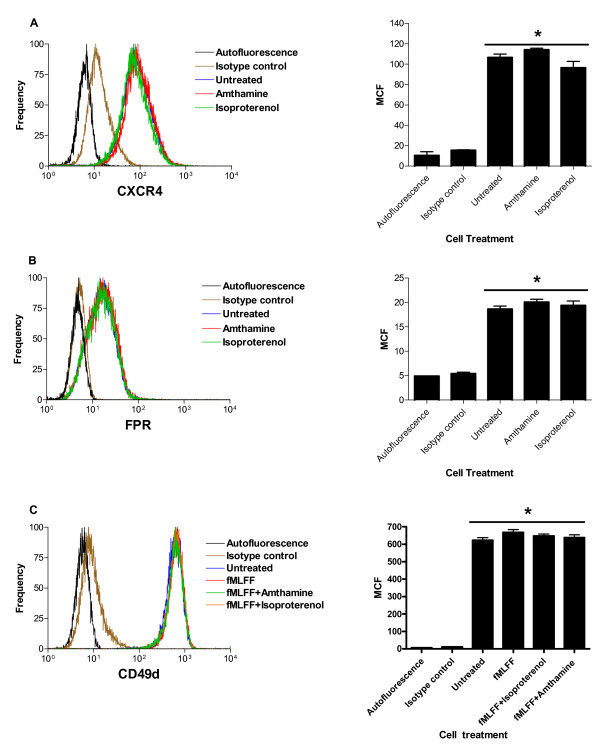
**Effect of Gα_s_-coupled receptor activation upon surface expression of CXCR4 receptor, formyl peptide receptor, and VLA-4**. U937 cells were treated with vehicle (untreated control), amthamine, isoproterenol, or sequentially with fMLFF, and vehicle (untreated control), amthamine or isoproterenol as described for experiments shown in Fig. 1 and 2. Next, cells were placed on ice and stained with anti-CXCR4 antibodies, A, anti-FPR antibodies, B, or CD49d antibodies, C. Histograms and bar graphs of mean channel fluorescence (MCF) ± SEM (n = 3) for unstained cell (autofluorescence), nonspecific binding (isotype control), treated with vehicle (untreated), and treated with different ligands are shown. One representative experiment out of two experiments is shown. * indicates means are not significantly different (P > 0.05) as calculated by one-way ANOVA analysis using GraphPad Prism software.

Next, in order to exclude the possibility that stimulation of Gα_s_-coupled receptors dramatically changes surface expression of Gα_i_-coupled receptors and VLA-4, U937 cells were treated with Gα_s_-coupled receptor agonists, and fMLFF in a manner that was identical to experiments described above (Fig. 1, 2). Then, cells were stained with anti-CXCR4, anti-FPR, and anti-CD49d antibodies (Fig. 3). No statistically significant changes in a surface expression were detected. Thus, the effect of down-regulation of integrin affinity is not related to the expression of Gα_i_-coupled GPCRs or VLA-4 integrin itself.

Earlier data suggest that FPR signalling typically requires occupancy of a fraction of the receptor sites [28]. In our experiments with saturating ligand concentration termination of signaling is expected to require a significant down-modulation of receptor expression within a few minutes. Furthermore, the ability of Gα_s_-coupled receptor antagonists to completely reverse the effect of Gα_s_-coupled receptor agonists (Fig. 2A, B, blue lines) indicates that integrin affinity down-regulation is triggered by the Gα_s_-coupled GPCRs activation rather than by Gα_i_-coupled deactivation. In addition, the use of the non-desensitizing FPR mutant emphasizes the role of Gα_s_-coupled signalling in the down-regulation of integrin affinity.

### Forskolin and dbcAMP mimic the effect of Gα_s_-coupled receptor signaling

**Figure 4:**
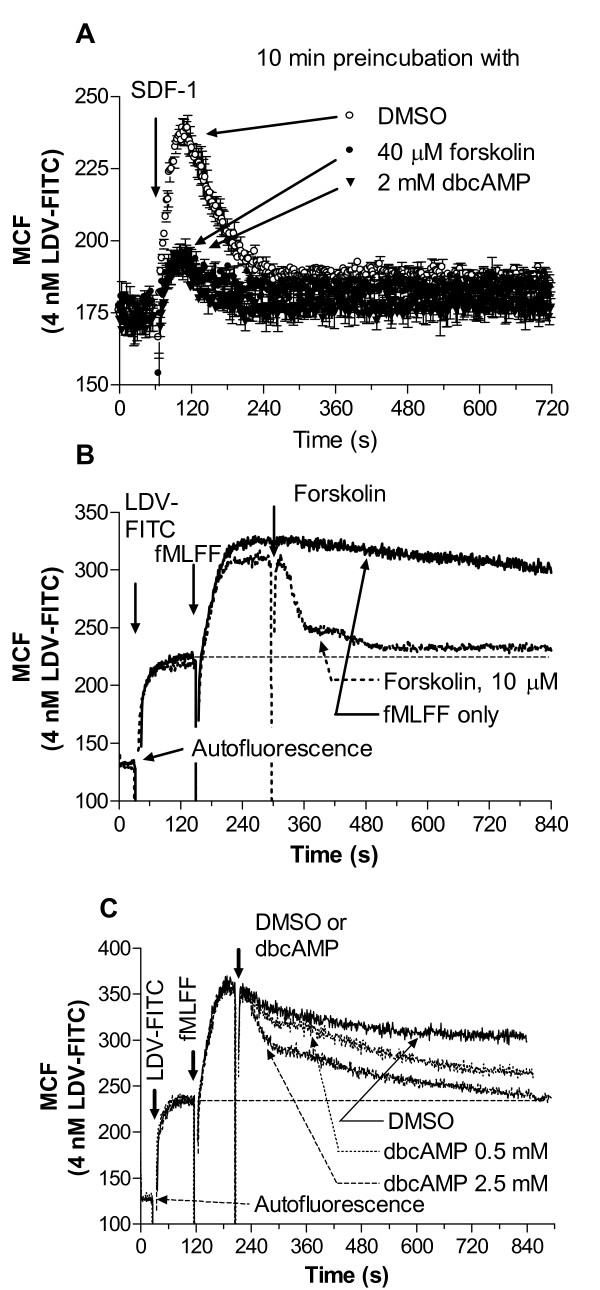
**Binding and dissociation of the LDV-FITC probe in response to cAMP agonists**. A, LDV-FITC probe binding and dissociation on U937 cells stably transfected with CXCR4 receptor plotted as mean channel fluorescence (MCF) versus time. Prior to SDF-1 addition, cells were preincubated with forskolin (40 μM), dibutyryl-cAMP (2 mM), or DMSO (control) for 10 min at 37°C. Next, cells were preincubated wth 4 nM LDV-FITC, and treated with SDF-1 (25 nM). Mean and standard error of mean are shown (n = 3). B, C, LDV-FITC probe binding and dissociation on U937 cells stably transfected with the non-desensitizing mutant of FPR plotted as mean channel fluorescence (MCF) versus time. After sequential additions of fluorescent LDV-FITC probe (4 nM), and fMLFF (100 nM), cells were treated with B, forskolin (10 μM), C, different concentration of dibutyryl-cAMP, or DMSO (control). The MCF value corresponding to cell autofluorescence is indicated by the horizontal arrow. One representative experiment out of three experiments is shown.

Gα_s_-coupled receptors are well known to activate adenylyl cyclase, which increases the concentration of intracellular cyclic adenosine monophosphate (cAMP). Also, treatment of U937 cells with β-adrenoceptor agonist induced a several fold elevation of intracellular cAMP concentration [29]. Therefore, we have tested whether activation of adenylyl cyclase by forskolin or dibutyryl cAMP would mimic the effects of Gα_s_-coupled receptors activation on VLA-4 affinity regulation. We found that preincubation of U937 cells with either compound abolished VLA-4 affinity up-regulation induced through CXCR4 receptor (Fig. 4A). We also detected affinity down regulation in real-time after activation of U937 cells through a non-desensitizing mutant of FPR (Fig. 4B,C). It is worth noting that the overall kinetics of the LDV-FITC ligand binding and dissociation in these cases was comparable to the case of Gα_s_-coupled receptor activation (compare Fig. 1 and Fig. 4). Thus, our data suggest that the down regulation of VLA-4 affinity after Gα_s_-coupled receptor activation is modulated by the increased concentration of cAMP.

### Signaling through Gα_s_-coupled receptors does not block VLA-4 unbending (extension)

**Figure 5:**
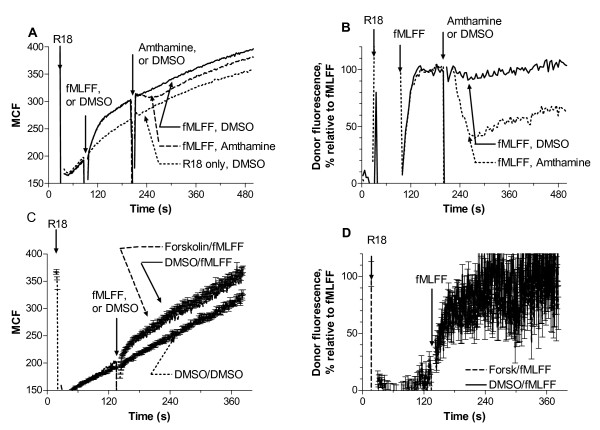
**Energy transfer, on U937 cells stably transfected with the non-desensitizing mutant of FPR, between the LDV-FITC donor probe and octadecylrhodamine (R18) acceptor probe**. Experiments were conducted as described under "Methods", and in [18,19]. A, Cells were preincubated at 37°C with 100 nM LDV-FITC probe to saturate high and low affinity sites. Next, LDV-FITC fluorescence was quenched after addition of octadecyl rhodamine (R18, 10 μM). Then, cells were activated by addition of fMLFF (100 nM). Data are plotted as MCF *versus *time for three conditions: quenched and then activated by Gα_i_-coupled receptor agonist (fMLFF (solid line)), quenched, then activated by Gα_i_-coupled receptor agonist (fMLFF) and then activated by Gα_s_-coupled receptor agonist (amthamine, 500 nM (dashed line)), and quenched only (R18 only, DMSO vehicle, dotted line). B, Data from panel A were re-plotted by subtracting the baseline data (R18 only) from activated cell data. The data are normalized assuming that average MCF value for FPR activated cells is equal to 100%; therefore, the Y-axis is labeled as "Donor fluorescence, % relative to fMLFF". As shown previously, activation of the cells transfected with the non-desensitizing mutant of FPR [18] led to the rapid unquenching of the FITC signal, which was interpreted as a change in the distance of closest approach between the VLA-4 ligand binding site occupied by LDV-FITC and the membrane surface (so called unbending or extension of the integrin molecule, see Fig. 1 in [18], or Fig. 5C in [19]). Curves are means out of five independent runs calculated on a point-by-point basis. C, Cells were preincubated for 5 min at 37°C with 10 μM of forskolin or DMSO (control). Next, cells were stained with 100 nM LDV-FITC probe and fluorescence was quenched after addition of octadecyl rhodamine (R18, 10 μM). Cells were activated by addition of 100 nM fMLFF, or DMSO (control). Notice the absence of the difference between forskolin treated (forskolin/fMLFF) and DMSO treated (DMSO/fMLFF) cells after addition of fMLFF. Mean and standard error of mean are shown (n = 3). D, Data from panel C were re-plotted as described for panel B.

A second aspect of integrin activation through "inside-out" signaling is a large conformational change, which results in the molecular unbending (extension) of the molecule, and has been associated with cell rolling [30]. The unbent integrin conformation results in faster cell aggregation rates, potentially because of the accessibility of the VLA-4 binding site [20]. Moreover, according to our recent findings, VLA-4 molecular extension is regulated by a mechanism, independent of a regulation of the affinity for the ligand [20]. Therefore, we studied the effect of Gα_s_-coupled receptor signaling on integrin extension using the FRET based real-time unbending assay (Fig. 5) [18-20].

Activation of U937 cell through a non-desensitizing mutant of FPR results in the rapid and sustained unquenching of the fluorescent signal, which is interpreted as a change in the distance of closest approach between the surface of the plasma membrane and the headgroup of the integrin molecule (molecular unbending) (see Methods and Fig. 1 in [18], or Fig. 5 in [19]). Addition of Gα_s_-coupled receptor ligands resulted in a rapid decrease of the fluorescent signal to about 40–50% of the control within ~60 seconds. Thereafter, the fluorescent signal slowly recovered back to ~70–90% of the control at the end of the experiment for the case of histamine H2 receptor simulation (Fig. 5A,B). The recovery phase was less definitive for the case of β2 adrenergic receptor stimulation (data not shown). Thus, the overall kinetics of the affinity change as detected by LDV-FITC probe binding and dissociation was entirely different from the kinetics of the molecular unbending as detected using FRET (compare Figs. 1, 2, 4 and Fig. 5A,B). In the case of the affinity change it takes up to 10 min to reach "full deactivation". The signal returns to the baseline value at 200–800 s after the beginning of the experiment, and no significant recovery was observed at this time scale (see Fig. 1B, Fig. 2A,B, Fig. 4B,C). In contrast, for the FRET signal, the response was maximal at ~1 min after Gα_s_-coupled receptor activation, and the signal did not return back to the baseline as in the case of the affinity change. This partial quenching result does not distinguish whether all VLA-4 molecules were still partially unbent (intermediate extended state) or whether a fraction of the VLA-4 molecules was still fully extended, while the rest of the integrins returned back to the bent conformation. However, the different kinetics of the FRET change and LDV-FITC binding support the idea that unbending and affinity are regulated though different signaling mechanisms [20].

Next, to study the effect of a sustained elevation of cAMP concentration upon integrin unbending we preincubated U937 with forskolin (10 μM for 5 min). This treatment was sufficient to completely inhibit (Fig. 4A) or reverse (Fig. 4B) the effect of Gα_i_-coupled receptors activation upon VLA-4 affinity up-regulation. However, we could not find any statistically significant difference in the FRET based real-time unbending assay between forskolin treated and DMSO (vehicle) treated cells (Fig. 5C,D). Thus, a sustained elevation of cAMP by forskolin had no effect upon VLA-4 unbending after Gα_i_-coupled receptor activation.

### The effect of Gα_s_-coupled receptor on VLA-4 activation is independent of Ca^2+^-signaling

**Figure 6:**
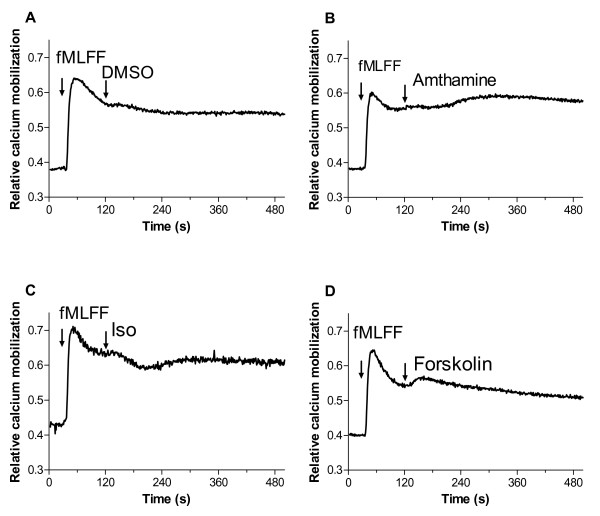
**Kinetics of intracellular Ca^2+ ^response, detected using Indo-1, AM, in U937 cells stably transfected with the non-desensitizing mutant of FPR in response to activation by Gα_i_/Gα_s_-coupled receptor agonists**. Experiments were conducted as described under "Methods". A, Control cells loaded with Indo-1 were treated with fMLFF (100 nM) and DMSO. B, Cells were sequentially treated with fMLFF (100 nM) and amthamine (500 nM); C, cells were treated with fMLFF (100 nM) and isoproterenol (100 nM); D, cells were treated with fMLFF and Forskolin (40 μM). One representative experiment out of three experiments is shown.

Conformational unbending of VLA-4 is dependent on the elevation of cytosolic Ca^2+ ^concentration, while affinity up-regulation can be observed in the absence of Ca^2+ ^increase [20]. Therefore, we tested whether in our model system Gα_s_-coupled receptor activation would significantly change intracellular Ca^2+^-concentration (Fig. 6). Activation through a non-desensitizing mutant of FPR resulted in sustained elevation of intracellular Ca^2+^. None of the Gα_s_-coupled receptor agonists significantly lowered the intracellular Ca^2+ ^concentration. In fact, we have observed a small increase of intracellular Ca^2+ ^after amthamine treatment (Fig. 6B). Thus, activation of Gα_s_-coupled receptors decreased VLA-4 affinity without significantly changing (decreasing) intracellular Ca^2+^-concentration.

These data additionally support the finding that the affinity state of the binding pocket is largely independent of cytoplasmic Ca^2+ ^level (Fig. 2 in [20]). Furthermore, partial quenching of the FRET signal (Fig. 5B) together with elevated intracellular Ca^2+ ^(Fig. 6) is analogous to the previously described condition, which arises several minutes after wild type Gα_i_-coupled receptor activation. There, the affinity state was already low because of rapid receptor desensitization, while VLA-4 integrins remained partially extended, as detected in the FRET based assay (see Fig. 7 in [20]).

**Figure 7:**
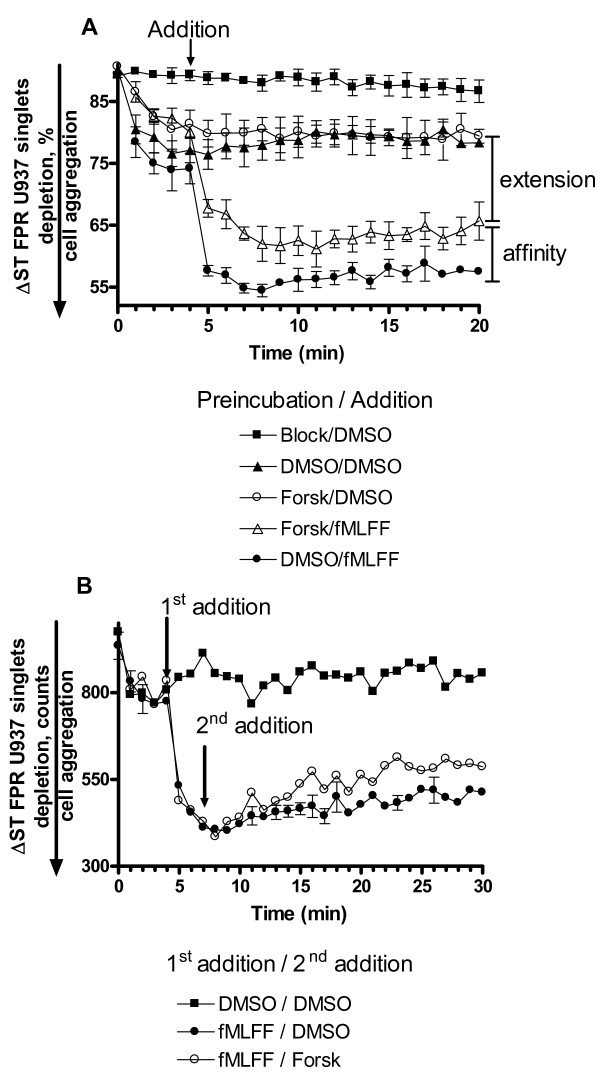
**Changes in cell adhesion between U937 FPR (ΔST) and VCAM-1-transfected B78H1 cells at resting state and in response to receptor stimulation**. *A*, U937 FPR (ΔST) cells were preincubated for 10 min at 37°C with forskolin (20 μM), or DMSO (vehicle). Cells were stimulated (arrow) with fMLFF (100 nM), or DMSO (vehicle). Data are plotted as % of U937 FPR (ΔST) singlets *versus *time. Five different experimental conditions are shown: Block/DMSO (preincubation with blocking 2 μM LDV small molecule (addition of DMSO, 1 μl), DMSO/DMSO, resting state (preincubation with DMSO/addition of DMSO), Forsk/DMSO (preincubation with forskolin/addition of DMSO), Forsk/fMLFF (preincubation with 20 μM forskolin/stimulation with 100 nM fMLFF), DMSO/fMLFF (preincubation with DMSO, stimulation with 100 nM fMLFF). The non-desensitizing FPR mutant is used to maintain VLA-4 in a state of constant high affinity. Mean and standard error of mean of three independent experiments are shown (n = 3). Bars on the right side of the panel indicate a relative contribution of affinity and extension in the overall cell adhesion. B, Effect of forskolin addition on adhesion in real-time. Cells were stimulated with fMLFF, or DMSO (1^st ^addition). Next, forskolin (20 μM) was added. Representative experiment out of two experiments is shown.

### The effect of Gα_s_/cAMP signaling pathway activation upon cell adhesion

Next, to study the implications of the Gα_s_-coupled signaling pathway on VLA-4 dependent cell adhesion we utilized a VLA-4/VCAM-1-specific real-time cell adhesion assay. The specificity of cell aggregation was tested using anti-α_4 _integrin mAb (HP2/1) [16] as well as the unlabeled LDV small molecule that completely blocked cell aggregation ([20] and Fig. 7A, solid squares). Prior to mixing, pre-stained (red, PKH26GL) U937 cells, stably transfected with non-desensitizing mutant of FPR, were treated with forskolin (20 μM, 10 min, 37°C). This treatment was sufficient to fully block/reverse the effect of Gα_i_-coupled receptors on the VLA-4 affinity change (Fig. 4A,B). However, it was insufficient to completely prevent rapid cellular aggregation induced by fMLFF (Fig. 7A, compare open and solid triangles after 5 min). A decrease in the number of free singlets in the media (singlets depletion) results from an increase in the number of aggregates, i.e. aggregation. It is worthwhile noting that we were also able to detect a statistically significant difference between forskolin treated and vehicle (DMSO) treated cells after fMLFF activation (Fig. 7A, compare open triangles and solid circles). Similar data were obtained using Gα_s_-coupled receptor activation (data not shown). This suggests that the cAMP pathway was able to reduce the potential of cells to form aggregates in response to Gα_i_-coupled receptor activation.

Since Gα_s_/cAMP signaling pathway blocks only one aspect of integrin activation, the affinity state of VLA-4 binding pocket, the difference between forskolin treated/fMLFF activated (open triangles) and untreated/fMLFF activated samples (solid circles) appears to be attributable to the impact of the affinity change on cell aggregation. The difference between unstimulated cells (solid triangles) and forskolin pretreated cells after fMLFF stimulation (open triangles) appears to be attributable to molecular unbending (extension).

The effect of Gα_s_/cAMP signaling pathway activation upon cell aggregation can be also observed in real-time (Fig. 7B). Addition of forskolin to cellular aggregates formed after fMLFF activation resulted in an increase in the number of free U937 singlets (Fig. 7B, compare solid and open circles after second addition). An increase in the number of free singlets in the media is interpreted as a decrease in a number of aggregates, i.e. disaggregation. Thus, rapid down regulation of VLA-4 affinity through Gα_s_/cAMP signaling pathway plays an important role in the regulation of VLA-4/VCAM-1 specific cell adhesion.

### VLA-4 affinity down-modulation can be detected on peripheral blood monocytes

**Figure 8:**
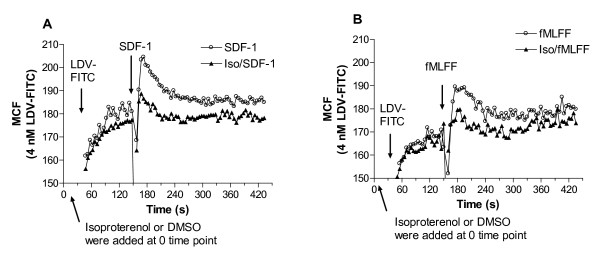
**Binding and dissociation of the LDV-FITC probe in response to Gα_s_-coupled receptor agonist**. Experiments were conducted as described under "Methods". A, LDV-FITC probe binding and dissociation on peripheral blood monocytes plotted as mean channel fluorescence (MCF) versus time. The experiment analogous to the one shown in Fig. 1A involved sequential additions of Gα_s_-coupled receptor ligand (isoproterenol (100 nM)) or DMSO (control), next, fluorescent LDV-FITC probe (4 nM, below saturation, added 2 min prior to addition of Gα_i_-coupled receptor ligand). Cells were activated with A, SDF-1 (25 nM, arrow), or B, fMLFF (100 nM, arrow). Rapid and reversible binding of the probe reflects a transient VLA-4 affinity change. Curves are means out of four independent runs calculated on a point-by-point basis. One representative experiment out of two independent experiments for each ligand is shown. Experiments shown in the different panels were performed using different instruments, and therefore MCF values are not identical.

Finally, to confirm that VLA-4 affinity down regulation can be detected in primary cells, we have used purified human peripheral blood monocytes. Monocytes express CXCR4 and FPR receptors. Rapid and transient binding of LDV-FITC can be observed in response to cell activation through these GPCRs [31]. Because, monocytes also express the β_2_-adrenergic receptor, we have used isoproterenol as Ga_s_-coupled receptor ligand. Experiments were performed in a manner identical to Fig. 1A. Human peripheral blood monocytes pretreated for 2.5 min with saturating isoproterenol had significantly reduced amplitude of LDV-FITC binding response after addition of SDF-1 (Fig. 8A), and fMLFF (Fig. 8B). In addition, pretreatment with isoproterenol affected the resting monocytes, resulting in lower LDV-FITC binding. These results were very similar to U937 cells (compare Fig. 1A and Fig. 8A). Thus, the effect of Gα_s_-dependent affinity down regulation of VLA-4 can be observed on human peripheral blood monocytes.

As shown previously, the number of α_4_-integrin molecules detected on peripheral blood cells is significantly lower than on the cell lines, which represents a less-differentiated phenotype. On peripheral blood human monocytes we have detected only ~6,000 sites. For U937 cells, we estimated about 40,000–100,000 sites [6]. Therefore, the overall signal from LDV-FITC on monocytes is ~10 fold lower than for U937 cells.

## Discussion

### LDV-FITC probe

The VLA-4 specific fluorescent probe was based on a highly specific α_4_β_1_-integrin inhibitor BIO1211, which contains the Leu-Asp-Val (LDV) ligand binding motif from the alternatively spliced connecting segment-1 (CS-1) peptide of cellular fibronectin [10,32]. Because carboxy-terminal amino acids did not affect ligand binding affinity (see Table 1 in [32]), they were replaced with Pro-Ala-Ala-Lys-FITC. This resulted in a fluorescent probe that we used to study VLA-4 integrin affinity/conformational changes in a conventional flow cytometer. One of the major advantages of this probe is that because of its low molecular weight, diffusion-limited rates of binding allow binding of the probe on the time scale relevant to dynamic cell responses. Therefore, this probe can be used to detect rapid and transient VLA-4-integrin affinity changes in real-time on live cells [6,16,19,20]. We have also used this probe to develop a FRET based assay for the detection of VLA-4 unbending (extension) in real-time after inside-out activation through GPCRs [18-20].

### VLA-4 affinity, extension, and cell adhesion

Previously, we established that affinities of the probe and native VLA-4 ligand (VCAM-1) vary in parallel when the integrin affinity state (natural lifetime) is modulated [16]. This affinity modulation results in a rapid change in cell adhesion efficiency. Moreover, molecular dissociation rates and cellular disaggregation rates in the absence of shear are comparable, suggesting that only few bonds are needed to form cellular aggregates. Therefore, we proposed that the increased aggregation is driven by integrin affinity changes, and not by clustering of the molecules or by increased bond numbers [17]. Moreover, using GPCR-transfected cells, we showed that the time course of avidity changes in response to the Gα_i_-coupled receptor activation coincides with the time course of the affinity changes. Affinity up-regulation led to rapid cell aggregation [6,16], and down regulation of affinity and extension resulted in the cells disaggregation [20].

Here we show that in addition to affinity up-regulation, the affinity of the VLA-4 binding pocket can be actively down-regulated, and this down regulation is in response to the Gα_s_-coupled receptors/cAMP signaling pathway. Moreover, affinity down regulation can be seen under continuous signaling conditions, after cell activation through a non-desensitizing mutant of Gα_i_-coupled GPCR. This mutant is known to produce normal Gα_i_-coupled signaling, but it cannot be phosphorylated, and thus it does not desensitize and internalize [33]. As the affinity of the VLA-4 binding pocket regulates the life-time of the VLA-4/VCAM bond [17], down regulation of integrin affinity would decrease the lifetime of the aggregate, and possibly reduce the transition to firm adhesion.

Another aspect of integrin activation is unbending (or extension) of the molecule [12,34]. Here we show that VLA-4 unbending was not inhibited by the Gα_s_/cAMP signaling pathway. In fact, even in the presence of a high concentration of forskolin, which blocked the VLA-4 affinity change completely, cells were able to respond to fMLFF activation by rapidly aggregating. This increase in cell adhesion is interpreted to arise solely from the unbending of the integrin molecule. Thus, for the first time in this model system we have established conditions under which cell adhesion was up-regulated exclusively due to the extension of the integrin molecule.

The discovery, that affinity of the integrin binding pocket and the molecular unbending are regulated by two different signalling pathways and modulate lifetime of adhesive interaction and cell capture efficiency, has already been documented [20]. We proposed that the low affinity unbent conformation represents an ideal rolling conformation (see Fig. 10B in [20]). We postulated that "several different proteins could participate in the "inside-out" activation of the integrins; some of them would regulate affinity, others – the conformational unbending" [20]. It is possible that affinity regulation by Gα_s_/cAMP signalling is directly related to Epac/Rap1 pathway, which is reported to affect integrin dependent cell adhesion [35]. For integrin unbending the molecular mechanism is still unclear. However, as shown previously, VLA-4 molecular unbending requires intracellular Ca^2+ ^increase [20]. Current data showed that activation via Gα_s_-coupled GPCRs did not significantly decrease intracellular Ca^2+ ^nor affect molecular unbending. This supports our current model of integrin conformation regulation in response to "inside-out" signalling.

### Active down regulation of VLA-4 affinity as a possible mechanism of cell trafficking

#### Cancer cell metastasis, role of NK cells

Tumor cell migration is essential in metastasis. As with hematopoietic progenitor cells, release and homing of certain cancer cells require adhesion molecules and Gα_i_-coupled GPCRs (such as VLA-4, CXCL12/CXCR4) [7]. Since human tissue microarrays showed that 70–90% of breast, colon, and prostate carcinoma tissues express the β2-adrenergic receptor [36], these cells may possess the extracellular signaling machinery needed for Gα_i_/Gα_s_-coupled to regulate cell adhesion. It has already been shown that β2-adrenergic receptor agonist activates, and β2-receptor antagonist blocks migration of prostate, colon and breast carcinoma cells in vitro [37-39]. In vivo, a β2-adrenergic receptor agonist significantly increased lymph node metastases, while an antagonist inhibited this effect, tumor growth was not affected by either treatment [36]. Thus, it is possible that down regulation of integrin affinity would significantly change the adhesive properties of tumor cells, facilitate cell migration/de-adhesion and lymphatic drainage, and thereby alter their overall metastatic potential.

NK cells play an important role in tumor cell clearance and resistance of organs to metastases. To effectively kill tumor cells, NK cells must localize at tumor sites. The VLA-4/VCAM-1 interaction is critical for recruitment of NK cells into lung, liver, and tumor sites [40]. As noted above, VLA-4 on NK cells exhibits constitutively high affinity for VCAM-1 as opposed to the low affinity state of VLA-4 on other circulating leukocytes [41,42]. Under stress, or treatment with β-adrenergic receptor agonist, a rapid rise in blood NK cells has been attributed to a reduction of their adherence to endothelia [43-45]. Since up-regulation of integrin affinity is essential for firm adhesion on vascular endothelia [9], affinity down-modulation would dramatically affect proper cell localization or recruitment. Affinity down-regulation could account for the phenomenon of β-adrenoceptor- or catecholamine-dependent suppression of NK cell activity, and a decrease in the overall resistance of an organism to metastasis [46].

#### Selective recruitment of leukocyte subsets

The multistep paradigm of leukocyte recruitment, where the interplay between selectins, chemokines, GPCRs, and integrins determines the specificity of leukocyte recruitment and trafficking, is broadly accepted [11,47-49]. GPCRs, through Gα_i _coupled inside-out signaling pathways, rapidly activate integrins, and trigger integrin dependent adhesion. Recently, it has been shown that in addition to pro-adhesive Gα_i_-coupled GPCRs, anti-adhesive Gα_s_-coupled receptors can modulate leukocyte transendothelial migration in vitro and recruitment and homing in vivo ([11] and references therein). That rapid affinity down-regulation can account for this modulation enriches our current understanding of selective recruitment of leukocyte subsets. It is worth noting that the same ligand can induce a pro-adhesive signal in one cell type (Gα_i_-coupled), and at the same time provide an anti-adhesive signal in another cell type (Gα_s_-coupled). For example, the H4 histamine receptor, selectively expressed on mast cells and eosinophils, is Gα_i_-coupled, and could serve as a chemotactic receptor for these cell types [50]. The Gα_s_-coupled H2 histamine receptor expressed on lymphocytes, macrophages and monocytes [51-53] could provide an anti-adhesive signal. Thus, active down-regulation of integrin affinity through Gα_s_-coupled GPCRs might be an essential mechanism regulating trafficking and selective recruitment of different leukocyte subsets.

## Methods

### Materials

The VLA-4 specific ligand [6,16,18] 4-((N'-2-methylphenyl)ureido)-phenylacetyl-L-leucyl-L-aspartyl-L-valyl-L-prolyl-L-alanyl-L-alanyl-L-lysine (LDV containing small molecule), and its FITC-conjugated analog (LDV-FITC) were synthesized at Commonwealth Biotechnologies. Octadecyl rhodamine B chloride (R18), and Indo-1, AM were from Invitrogen. Restriction enzymes were purchased from New England BioLabs. Human recombinant CXCL12/SDF-1α was from R&D Systems. Mouse anti-human CD14/R-PE antibodies, clone UCHM1, and isotype control mouse IgG2a/R-PE, clone RPC 5 were from Ancell Corp. PE mouse anti-human CXCR4 (CD184 PE) antibodies, clone 12G5, isotype control (mouse IgG2a κ PE) clone G155-178, PE mouse anti-human formyl peptide receptor, clone 5F1, isotype control (mouse IgG1 κ PE) clone MOPC-21, PE mouse anti-human CD49d (α_4_-integrin subunit PE) clone 9F10 were purchased from BD Biosciences and used according to manufacturer instructions. All other reagents were from Sigma-Aldrich. Stock solutions were prepared in DMSO, at concentrations ~1000 fold higher than the final concentration. Usually, 1 μl of stock solution was added to 1 ml of cell suspension yielding a final DMSO concentration of 0.1%. Control samples were treated with equal amount of pure DMSO (vehicle).

### Cell Lines and Transfectant Construct

The human histiocytic lymphoma cell line U937 was purchased from ATCC. Wild type CXCR4 (CD184) receptor stably transfected U937 cells, and site-directed mutants of the FPR (non-desensitizing mutant of FPR ΔST) in U937 cells were prepared as described [54] and were a gift of Dr. Eric Prossnitz (University of New Mexico). High receptor expressing cells were selected using the MoFlo Flow Cytometer (DakoCytomation). Cells were grown at 37°C in a humidified atmosphere of 5% CO_2 _and 95% air in RPMI 1640 (supplemented with 2 mM L-glutamine, 100 units/mL penicillin, 100 μg/mL streptomycin, 10 mM HEPES, pH 7.4, and 10% heat inactivated fetal bovine serum). Cells were then harvested and resuspended in 1 ml of HEPES buffer (110 mM NaCl, 10 mM KCl, 10 mM glucose, 1 mM MgCl_2_, 1.5 mM CaCl_2 _and 30 mM HEPES, pH 7.4) containing 0.1% HSA and stored on ice. The buffer was depleted of lipopolysaccharide by affinity chromatography over polymyxin B sepharose (Detoxigel; Pierce Scientific). Cells were counted using the Coulter Multisizer/Z2 analyzer (Beckman Coulter). For experiments, cells were suspended in the same HEPES buffer at 1 × 10^6 ^cells/ml and warmed to 37°C. Alternatively, cell were resuspended in serum/dye-free warm RPMI (37°C) and used immediately.

### Purification of human peripheral blood monocytes

Human peripheral blood mononuclear leukocytes were isolated by density gradient centrifugation. Venous blood anticoagulated with heparin or EDTA was obtained from healthy volunteers, and the buffy coat was layered over Ficoll Hypaque (Amersham Biosciences), and centrifuged at 1162 *g *to obtain mononuclear leukocytes. Leukocytes were washed in 2 mM EDTA in PBS without calcium/magnesium chloride (Gibco), resuspended in RPMI 1640 supplemented with 10% heat-inactivated fetal bovine serum and used immediately. Monocytes were purified from mononuclear leukocytes using AutoMACS automated magnetic cell sorter (Miltenyi Biotec) by negative depletion using magnetic beads with anti-human CD3, CD16, CD19, CD56 specific antibodies (Miltenyi Biotec) according to manufacturer instructions. Purified monocytes were resuspended in HHB buffer supplemented with 0.1% HSA at 1 × 10^6 ^cells/ml and used within 1–3 hours. Monocyte purity was tested using anti-human CD14 PE antibodies (clone UCHM1, Ancell Corp.). For LDV-FITC binding experiments the population of cells was gated on forward vs. side scatter intensity based on CD14^+ ^cells.

### Kinetic Analysis of Binding and Dissociation of VLA-4 Specific Ligand

Kinetic analysis of the binding and dissociation of the LDV-FITC probe was described previously [6]. Briefly, cells (1 × 10^6 ^cells/ml) were preincubated in HEPES buffer containing 0.1% HSA at different conditions for 10–20 min at 37°C. Flow cytometric data were acquired for up to 1024 s at 37°C while the samples were stirred continuously at 300 rpm with a 5 × 2 mm magnetic stir bar (Bel-Art Products). Samples were analyzed for 30–120 s to establish a baseline. The fluorescent ligand was added and acquisition was re-established, creating a 5–10 s gap in the time course. For real-time affinity activation experiments, 4 nM LDV-FITC was added after establishing a baseline for unstained cells marked on figures as "autofluorescence". Then, data were acquired for 2–3 minutes, and cells were treated with different GPCR ligands at saturating concentration (10 times or higher than K_d_). In several experiments cells were treated sequentially with two or more different compounds. Acquisition was re-established, and data were acquired continuously for up to 1024 s. The concentration of the LDV-FITC probe used in the experiments (4 nM) was below the dissociation constant (K_d_) for its binding to resting VLA-4 (low affinity state, K_d_~12 nM), and above the K_d _for physiologically activated VLA-4 (high affinity state, K_d_~1–2 nM) [6]. Therefore, the transition from the low affinity to the high affinity receptor state led to increased binding of the probe (from ~25% to ~70–80% of receptor occupancy, as calculated based on the one site binding equation), which was detected as an increase in the mean channel fluorescence (MCF). For kinetic dissociation measurements, cell samples were preincubated with the fluorescent probe (25 nM), treated with excess unlabeled LDV containing small molecule (2 μM) and the dissociation of the fluorescent molecule was followed. The resulting data were converted to MCF *versus *time using FCSQuery software developed by Dr. Bruce Edwards (University of New Mexico).

GPCR ligand concentrations were chosen based upon known binding affinities [55-60]. Ligands were used at the concentrations of two or more fold higher than respective dissociation constants (Kd). This results in more than 66% coverage of the receptor sites. For Gα_s_-coupled receptor binding site competition experiments (Fig. 2A,B) lower agonist concentrations were chosen to limit potential rebinding after addition of a large excess of antagonist (20 fold or more). The concentrations of antagonists were chosen based on their respective affinities, to cover more than 99% of receptor binding sites (200 fold greater than Kd). By themselves, these high concentrations of Gα_s_-coupled receptor antagonists did not affect cells activation after stimulation by Gα_i_-coupled receptor agonists (see Fig. 2B, black line).

### FRET Detection of VLA-4 Unbending (Extension)

The fluorescence resonance energy transfer (FRET) assay used the LDV-FITC probe as a donor, which specifically binds to the α_4_-integrin headgroup, and octadecyl rhodamine B (R18) as an acceptor incorporated into the plasma membrane was previously described in [18] and validated in several publications [19,20]. Briefly, U937 cells stably transfected with CXCR4 or the non-desensitizing mutant (ΔST) of the formyl peptide receptor [33,61], were preincubated with 100 nM LDV-FITC probe in HEPES buffer containing 1.5 mM CaCl_2_, 1 mM MgCl_2_, and 0.1% HSA at 37°C. Alternatively, 100 nM LDV-FITC was added after establishing a baseline for unstained cells (autofluorescence). Next, samples were analyzed for 1–3 minutes to establish a baseline for 100 nM LDV-FITC, and then a saturating amount of octadecyl rhodamine B (R18, 10 μM final) was added to yield maximal quenching of donor fluorescence. 1–3 min after R18 was added; cells were treated with different GPCR ligands at saturating concentration (10 times or higher than K_d_). In several experiments cells were treated sequentially with two or more different compounds as in the LDV-FITC probe binding experiments. Donor intensities (FL1 in MCF units) were measured using a Becton-Dickinson FACScan flow cytometer at 37°C. Changes in the donor fluorescence intensity were interpreted as changes in the distance of closest approach between LDV-FITC ligand binding site on VLA-4 and the surface of the plasma membrane [18,19].

### Calcium Mobilization

Calcium mobilization in response to ligand stimulation was measured as described previously [61]. Briefly cells were harvested by centrifugation, washed, and resuspended at 5 × 10^6 ^cells/ml in HEPES buffer containing 1.5 mM CaCl_2_, 1 mM MgCl_2_. Next, cells were incubated with 5 μM Indo-1, AM for 30 min at 37°C, washed, and resuspended at 1 × 10^6 ^cells/ml in HEPES buffer, and stored on ice. Cells were allowed to equilibrate at 37°C for 2 min, stimulated with different GPCR ligands, and continuous fluorescence was monitored using a spectrofluorometer (Photon Technologies International). Excitation at 350 nm, detection at 405 nm and 490 nm. Fluorescence intensity ratio (405 nm/490 nm) was computed in real-time as a measure of intracellular Ca^2+^.

### Cell Adhesion Assay

The cell suspension adhesion assay has been described previously [16]. Briefly, U937/ΔST FPR stably transfected cells were labeled with red fluorescent PKH26GL dye, and B78H1/VCAM-1 transfectants were stained with green fluorescent PKH67GL dye (Sigma-Aldrich). Labeled cells were washed, resuspended in HEPES buffer supplemented with 0.1% HSA and stored on ice until used in assays. Control U937 cells were preincubated with the LDV-containing small molecule for blocking. Prior to data acquisition, cells were warmed to 37°C for 10 min separately and then mixed. During data acquisition, the samples were stirred with a 5 × 2-mm magnetic stir bar (Bel-Art Products, Pequannock, NJ) at 300 rpm and kept at 37°C. For stimulation, cells were treated with different GPCR ligands at saturating concentration (10 times or higher than K_d_) as described in "Kinetic Analysis of Binding and Dissociation of VLA-4 Specific Ligand". In several experiments cells were treated sequentially with two different compounds. The number of cell aggregates containing U937 adherent to B78H1/VCAM-1 (red and green cofluorescent particles) as well as a number of singlets (red or green fluorescent particles, FL2 and FL1 in FACScan flow cytometer) were followed in real time. As shown previously, cell aggregation as detected using cell aggregates (red and green cofluorescent particles) did not discriminate between doublets and higher order aggregates [17]. Therefore, the analysis of cell aggregation was done by using singlet cell depletion methodology [62]. The percentage of singlets was calculated as follows: singlets depletion, % = (number of singlets/(number of aggregates + number of singlets)) × 100. Experiments were done using a FACScan flow cytometer and Cell Quest software (Becton Dickinson, San Jose, CA). The data were converted to number of singlets/aggregates *versus *time using FCSQuery software developed by Dr. Bruce Edwards (University of New Mexico).

### Statistical analysis

Curve fits and statistics were performed using GraphPad Prism (GraphPad). Each experiment was repeated at least three times. The experimental curves represent the mean of two or more independent runs. SEM was calculated using GraphPad Prism.

## Abbreviations

cAMP (adenosine 3',5'-cyclophosphate), dbcAMP (N-6,2'-O-dibutyryladenosine 3',5'-cyclic monophosphate), fMLFF (N-formyl-L-methionyl-L-leucyl-L-phenylalanyl-L-phenylalanine, formyl peptide), FPR (formyl peptide receptor 1), FRET (fluorescence resonance energy transfer), GPCR (guanine nucleotide binding protein coupled receptor), HSA (human serum albumin), HEPES (4-(2-hydroxyethyl)-1-piperazineethanesulfonic acid), LDV containing small molecule (4-((N'-2-methylphenyl)ureido)-phenylacetyl-L-leucyl-L-aspartyl-L-valyl-L-prolyl-L-alanyl-L-alanyl-L-lysine), LDV-FITC containing small molecule (4-((N'-2-methylphenyl)ureido)-phenylacetyl-L-leucyl-L-aspartyl-L-valyl-L-prolyl-L-alanyl-L-alanyl-L-lysine-FITC), mAb (monoclonal antibody) LFA-1 (lymphocyte function-associated antigen-1, CD11a/CD18, α_L_β_2 _integrin), MCF (mean channel fluorescence, equivalent of mean fluorescence intensity), NK cell (natural killer cells), SDF-1 (stromal cell-derived factor-1, CXCL12), VCAM-1 (vascular cell adhesion molecule 1, CD106), VLA-4 (very late antigen 4, CD49d/CD29, α_4_β_1 _integrin).

## Authors' contributions

AC designed the study, carried out ligand binding experiments, cell adhesion studies, and wrote the manuscript. AW carried out β2-adrenergic receptor experiments, participated in study design. OA performed antibody staining experiments and helped with monocytes experiments. LAS contributed to the study concept, and participated in study design and coordination. All authors read and approved the final manuscript.
